# A systematic review of interventions to reduce HIV-related stigma and discrimination from 2002 to 2013: how far have we come?

**DOI:** 10.7448/IAS.16.3.18734

**Published:** 2013-11-13

**Authors:** Anne L Stangl, Jennifer K Lloyd, Laura M Brady, Claire E Holland, Stefan Baral

**Affiliations:** 1 Department of Global Health, International Center for Research on Women, Washington, DC, USA; 2 Department of Epidemiology, Johns Hopkins School of Public Health, Baltimore, MD, USA

**Keywords:** systematic review, HIV, stigma reduction, discrimination reduction, interventions, measurement

## Abstract

**Introduction:**

HIV-related stigma and discrimination continue to hamper efforts to prevent new infections and engage people in HIV treatment, care and support programmes. The identification of effective interventions to reduce stigma and discrimination that can be integrated into national responses is crucial to the success of the global AIDS response.

**Methods:**

We conducted a systematic review of studies and reports that assessed the effectiveness of interventions to reduce HIV stigma and discrimination between 1 January 2002 and 1 March 2013. Databases searched for peer-reviewed articles included PubMed, Scopus, EBSCO Host –CINAHL Plus, Psycinfo, Ovid, Sociofile and Popline. Reports were obtained from the www.HIVAIDSClearinghouse.eu, USAID Development Experience Clearinghouse, UNESCO HIV and AIDS Education Clearinghouse, Google, WHO and UNAIDS. Ancestry searches for articles included in the systematic review were also conducted. Studies of any design that sought to reduce stigma as a primary or secondary objective and included pre- and post-intervention measures of stigma were included.

**Results:**

Of 2368 peer-reviewed articles and reports identified, 48 were included in our review representing 14 different target populations in 28 countries. The majority of interventions utilized two or more strategies to reduce stigma and discrimination, and ten included structural or biomedical components. However, most interventions targeted a single socio-ecological level and a single domain of stigma. Outcome measures lacked uniformity and validity, making both interpretation and comparison of study results difficult. While the majority of studies were effective at reducing the aspects of stigma they measured, none assessed the influence of stigma or discrimination reduction on HIV-related health outcomes.

**Conclusions:**

Our review revealed considerable progress in the stigma-reduction field. However, critical challenges and gaps remain which are impeding the identification of effective stigma-reduction strategies that can be implemented by national governments on a larger scale. The development, validation, and consistent use of globally relevant scales of stigma and discrimination are a critical next step for advancing the field of research in this area. Studies comparing the effectiveness of different stigma-reduction strategies and studies assessing the influence of stigma reduction on key behavioural and biomedical outcomes are also needed to maximize biomedical prevention efforts.

## Introduction

More than two decades into the HIV epidemic, stigma and discrimination continue to hamper efforts to prevent new infections and engage people in HIV treatment, care and support programmes. Numerous studies have linked HIV-related stigma with refusal of HIV testing, non-disclosure to partners and poor engagement in biomedical prevention approaches [1–6]. Similarly, internalized stigma, which refers to the negative consequences that result when people believe that stigmatizing public attitudes apply to them [7, 8], is a well-established barrier to medication adherence [9–13]. In response to this evidence, stigma reduction is now a key priority in PEPFAR's Blueprint for Achieving an AIDS-Free Generation [14] and UNAIDS’ HIV investment framework [15].

The recent shift in the global AIDS response to biomedical prevention will require acceptance and uptake of prevention approaches, such as voluntary medical male circumcision, pre-exposure prophylaxis and universal testing and treatment, at the population level [16, 17]. Effective interventions to reduce stigma and discrimination are crucial to the success of biomedical prevention [15, 18]. Such interventions need to be integrated into national responses and address the stigmatization process [19].

### Stigma conceptualizations and terminology

Stigma has been conceptualized from the perspective of both the individual and the society. According to Erving Goffman, stigma occurs when an attribute creates a deeply discrediting gap between who we think we are – our “actual social identity” – and how we are seen by others – our “virtual social identity” [20]. This gap creates a “spoiled identity” that cuts the stigmatized person “off from society and from himself, so that he stands as a discredited person against an unaccepting world” [20]. Building on Goffman's work, Link and Phelan described stigma as a harmful societal phenomenon – enabled by underlying social, political and economic powers – that begins when a difference is labelled, then is linked to negative stereotypes, leading to a separation of “us” from “them,” and finally to status loss and discrimination for those carrying the trait [21]. Deacon suggested that HIV-related discrimination be analyzed separately from stigma to explore the range of stigma-related disadvantages that may result from the stigmatization process, as well as positive responses such as resilience and activism [22].

The stigmatization process can be broken into specific domains, each of which can be addressed through programmatic and policy efforts [19, 23]. These domains are: drivers, facilitators, intersecting stigmas and manifestations of stigma [19]. Drivers are individual-level factors that negatively influence the stigmatization process such as: lack of awareness of stigma and its harmful consequences, fear of HIV infection through casual contact with people living with HIV (PLHIV), fear of economic ramifications or social breakdown due to HIV-positive family and community members, and prejudice and stereotypes towards PLHIV and key populations at highest risk of HIV infection [24–27]. Facilitators are societal-level factors that influence the stigmatization process either negatively or positively, including: protective or punitive laws, availability of grievance redressal systems, awareness of rights, structural barriers at the public policy level, cultural and gender norms, existence of social support for PLHIV, and power/powerlessness among PLHIV to resist and overcome the manifestations of stigma [19].

Drivers and facilitators combine to influence whether a stigma is applied to individuals or groups based on HIV status.

Intersecting, or layered, stigmas, refer to the multiple stigmas that people often face due to HIV status, gender, profession, migrancy, drug use, poverty, marital status, sexual and gender orientation [28–31]. Manifestations are the immediate results, mostly negative, of a stigma being applied to individuals or groups, including: anticipated stigma (fear of experiencing stigma if HIV status becomes known) [32], perceived stigma (perceptions about how PLHIV are treated in a given context) [33], internalized stigma [34], shame [35], experienced, or enacted, stigma (experiencing stigmatizing behaviours outside the purview of the law) [36, 37], discrimination (experiencing stigmatizing behaviours within the purview of the law) and resilience (ability to overcome threats to health and development after stigma is experienced) [18, 19]. Distinguishing between experienced stigma and experienced discrimination based on their legality informs the intervention strategies needed.

Individuals experience, internalize and/or perpetuate the manifestations of stigma [19]. Additionally, the social and structural environments in which individuals live and work influence the drivers and manifestations of stigma [38, 39], indicating the need for interventions that target multiple levels [40]. The socio-ecological framework [41], which recognizes that societal norms and structures influence individual attitudes and behaviours, identifies key levels at which stigma-reduction activities can be targeted: individual (knowledge, attitudes, skills), interpersonal (family, friends, social networks), organizational (organizations, social institutions, workplace), community (cultural values, norms, attitudes) and public policy (national and local laws and policies) [42].

### Previous reviews

Brown et al. [43] conducted the first global review of interventions to reduce HIV-related stigma in 2003. The authors articulated four intervention categories based on psychosocial conceptualizations of the stigmatization process that have remained applicable a decade later. The categories include:

1. information-based approaches (e.g., written information in a brochure),
2. skills building (e.g., participatory learning sessions to reduce negative attitudes),
3. counselling/support (e.g., support groups for PLHIV), and
4. contact with affected groups (e.g., interactions between PLHIV and the general public).

Most of the 22 studies reviewed attempted to increase the general public's tolerance or health providers’ willingness to treat PLHIV by changing individual-level fears, attitudes or behaviours. Two studies sought to improve coping strategies among PLHIV or key populations. The authors concluded that some stigma-reduction interventions appeared to work in the short term, but that more research was needed to understand the effectiveness of various intervention components, the scale and length of interventions required, and the gendered impacts [43].

The second review by Sengupta et al. in 2011 examined 19 HIV-prevention interventions that measured HIV stigma pre- and post-intervention, 11 of which had one or more components that directly targeted HIV stigma [44]. The review found that information, skills-building, counselling and PLHIV testimonials were associated with less stigmatizing attitudes among participants. The authors noted several gaps in the evidence base, including the poor quality of the majority of studies reviewed and the lack of standardized measurement [44].

### Current review

Our goal in the current systematic review was to obtain a more complete picture of the full range of intervention efforts and their effectiveness in interrupting the stigmatization process, minimizing negative manifestations of stigma and/or bolstering positive manifestations, such as resilience. An important distinction from previous reviews was the inclusion of search terms to capture discrimination-reduction interventions separately from stigma-reduction interventions. Another unique feature was the inclusion of structural and biomedical intervention categories.

Recent literature has focused on the role of structural and biomedical approaches in the prevention of HIV acquisition and transmission [45–48]. In the context of HIV-related stigma, structural approaches encompass activities aimed at removing, reducing or altering for the better structural factors that influence the stigmatization process, such as laws that criminalize HIV [49], hospital or workplace policies that institutionalize discrimination of PLHIV (e.g., labelling of beds, mandatory HIV testing prior to employment), or a lack of supplies to allow healthcare workers to practice universal precautions [40]. Structural approaches can also include efforts to ensure that grievance redressal systems and legal aid are available for PLHIV to seek justice if discriminated against [50, 51]. The emergence of structural interventions to reduce HIV-related stigma and discrimination is in direct response to the underlying power structures that enable the stigmatization process [21, 52]. The expansion of biomedical prevention approaches may influence HIV-related stigma, either positively, by normalizing HIV infection, or negatively, by leading to unwanted disclosure of sero-positive status and resulting discrimination [53, 54]. However, this relationship has yet to be explored quantitatively in the literature.

To identify interventions targeting HIV-related stigma and/or discrimination, we systematically reviewed peer-reviewed and grey literature. Our objectives were to document the stigma domains addressed, socio-ecological levels targeted, types of strategies employed to reduce stigma and discrimination, stigma-specific outcomes of these efforts and study quality.

## Methods

### Search strategy and selection criteria

This review followed PRISMA guidelines. Search terms included MESH or other associated terms for HIV cross-referenced with “stigma reduction” OR “discrimination reduction” (see Supplementary files). Databases for peer-reviewed articles included PubMed, Scopus, EBSCO Host – CINAHL Plus, Psycinfo, Ovid, Sociofile and Popline. Grey literature was obtained from the www.HIVAIDSClearinghouse.eu, USAID Development Experience Clearinghouse, UNESCO HIV and AIDS Education Clearinghouse, Google, WHO and UNAIDS. Ancestry searches of the 48 articles included in the review were also conducted.

Inclusion criteria included pre- and post-test data, clear descriptions of the intervention and sampling methods, and publication in English. We limited our search to articles published between 1 January 2002 and 1 March 2013 to exclude articles included in the Brown et al. review (2003) [43]. Studies of any design from any country that listed HIV stigma and/or discrimination reduction as a primary or secondary outcome were included. Studies were excluded if none of the intervention components aimed to reduce HIV stigma and/or discrimination. We did not exclude studies that lacked a clear description of the measures used or those that used non-validated measures, as historically these issues have been inconsistently addressed [36, 43, 44].

### Screening and data abstraction

Article citations were organized, uploaded and reviewed using the reference manager programme Endnote X5 from their respective databases. The title, author, journal and year of publication were then exported to an excel spreadsheet for title and abstract review. Articles were screened by two of three reviewers (JKL, LMB, CEH) to determine whether they included relevant information. If the article was deemed relevant by at least one reviewer, the abstract was retrieved. The same two reviewers screened the abstracts for relevant information. If at least one reviewer deemed the abstract relevant, or if the full text had to be obtained to determine if the abstract was relevant, the full text was reviewed. Discrepancies were discussed with a third senior reviewer (ALS) and consensus was reached as to whether or not to include the article. Data were abstracted using a standardized abstraction form (see Supplementary files). For studies that did not specify the validity or number of stigma measures used, the corresponding author was contacted. For measures coded as “not specified” (NS) in Table 1, we did not receive a response.

**Table 1:** Study and intervention characteristics, description of stigma measures and study findings from 48 studies

First author, publication date, country, study design^a^	Study population^b^	Sample	Intervention strategies^c^, intervention duration	Stigma domains^d^, socio-ecological levels^e^	Validated/un-validated, no. of items^f^	Results
Intervention strategy used						
Adam, 2011 [81], Canadian web-based, RXS	MSM	1942	I, 4 months	D, individual	Un-validated, 5 items	Stigma decreased
Al-Mazrou, 2005 [92], Saudi Arabia, QE/NC	Students (paramedical)	653	I, 1 year	D, individual	NS, NS	Stigma decreased
Bell, 2008 [72], South Africa, RCT	Students (primary), caregivers	557, 478	SB, 10 weekends, 90 minutes sessions	D, individual	Validated, NS	Stigma decreased
Esu-Williams, 2004 [77], Zambia, QE/C	Youth club members	60	SB, 3 years	D, individual	NS, NS	Stigma decreased
Li, 2011 [65], China, QE/C	Students (high school)	287	I, 8 sessions, 90 minutes	D, individual	Un-validated, 1 item	Stigma decreased
Maughan-Brown, 2010 [102], South Africa, RXS	Young adults	1074	B, 3 years	D, public policy	Un-validated, 8 items	Stigma increased
Nambiar, 2011 [69], India, QE/C	PLHIV	257	I, 14 days	M, individual	Un-validated, 36 items	Enacted stigma reduced. No change in felt or disclosure stigma
Neema, 2012 [99], Uganda, RXS	PLHIV	475	SB, 1 year, 6 months	F, organizational	NS, NS	Stigma decreased
Norr, 2012 [91], Chile, QE/C	HCWs	555	I, 8 sessions, 3 month F-U	D, individual	Un-validated, 7 items	Stigma decreased
Paxton, 2002 [66], Australia, QE/C	Students (secondary)	1397	C, 12 talks, 3 month F-U	D, individual	Validated, 15-item scale	Stigma decreased, but the impact was reduced after 3 months.
Sorcar, 2009 [67], India, QE/C	Students, (high school and college)	386	I, 3 stages, 1 year	D, individual	Un-validated, 17 items	Stigma decreased
Wang, 2009 [61], China, QE/NC	HCWs	69	SB, 10 days	D, individual	NS, NS	Stigma decreased
Intervention strategies used						
Bekele, 2008 [73], Ethiopia, QE/NC	Students, (high school)	373	I, SB, 8 hours	D, individual	Un-validated, 61 items	Stigma decreased
Biradavolu, 2012 [104], India, Pre- post- qualitative IDIs	FSW	55	ST, SB, 1 year, 5 months	D, M, organizational	N/A*	Stigma decreased
Boulay, 2008 [85], Ghana, RXS	Community members	5672	I, SB, 2 months	D, community	NS, 8 items	Stigma decreased
Brown, 2009 [74], South Africa, QE/C	Students, (university)	237	I, C, 3 weeks, 1 hour sessions	D, individual	Validated, 10-item scale	Stigma decreased
Deutsch, 2007 [82], USA, QE/C	Students (university)	77	I, SB, 2 sessions, 2 weeks	D, individual	Validated, 54-item scale	Stigma decreased
Denison, 2012 [79], Zambia, QE/C	Students (grade 8–9)	2133	I, SB, 1 month	D, organizational	Un-validated, 4 items	Stigma decreased
Ezedinachi, 2002 [87], Nigeria, RCT	HCWs	1552	I, SB, 30 workshops, 1 year F-U	D, individual	Un-validated, 14	Stigma decreased
Fakolade, 2010 [86], Nigeria, RXS	Community members	31,692	I, C, 4 years	D, community	NS, NS	Stigma decreased
Jurgensen, 2013 [80], Zambia, RCT	Community members	2607	CS, B, 2 years	D, public policy	Validated, 8-item scale	Stigma decreased in both intervention and control arm
Kaponda, 2009 [71], Malawi, QE/NC	HCWs	855	I, SB, 10, 90–120 minutes workshops	D, individual	NS, 2 items	Stigma decreased
Lau, 2005 [64], Hong Kong, QE/NC	Students, (grade 9–10)	1153	I, C, 2 weeks	D, individual	Un-validated, 19 items	Stigma decreased
Li, 2010 [60], China, RCT	Market workers	4510	I, SB, 2 years	D, community	Un-validated, 4 items	Stigma decreased
Norr, 2007 [76], Malawi, QE/NC	Teachers	328	I, SB, 6, 2-hour sessions	D, individual	Un-validated, 6 items	Stigma decreased
Richter, 2012 [103], Angola, Cameroon, Chad, Cote D'Ivoire, Equatorial Guinea, Kenya, Nigeria, QE/NC	Employees	993	I, SB, 15 sessions, 12–18 mos.	D, F, individual, organizational	Validated and un-validated, 11 items	Stigma decreased
Rimal, 2008 [70], Malawi, RXS	Community members	1771	I, C, 2 years	D, community	Un-validated, 14 items	Stigma decreased for those with high efficacy only; no change for those with low efficacy
Saad, 2012 [88], Nigeria, RCT	Students (university)	235	I, SB, 8-hour programme, 3 and 6-month F-U	D, community	Validated, 9-item scale	No change
Smith Fawzi, 2012 [89], Haiti, QE/NC	HIV+youth and their caregivers	168, 130	I, SB, 1 year	M, interpersonal	Validated, 22-item scale	Stigma decreased
Tshabalala, 2011 [100], South Africa, QE/C	PLHIV	20	I, SB, 8 sessions	D, M, individual	Validated, 16-item scale	Internalized stigma decreased. No change in enacted stigma
Williams, 2006 [62], China, QE/NC	HCWs	208	I, SB, 5-day workshop	D, individual	Validated, 34-item scale	Stigma decreased
Wu, 2008 [68], China, QE/C	HCWs	138	I, SB, 1, 4-hour session, 3 and 6-month F-U	D, individual	Un-validated, 3 items	Stigma decreased
Yiu, 2010 [68], Hong Kong, QE/NC	Students, (nursing)	89	I, C, 50-minute lecture, 6-week F-U	D, individual	Un-validated, 15 items	Stigma decreased
Young, 2010 [90], Peru, RCT	Community members	3049	I, SB, 24 months	D, community	Un-validated, 5 items	Stigma decreased for men, not for women
Intervention strategies used						
Apinundecha, 2007 [101], Thailand, QE/C	PLHIV, caregivers, and community leaders	425	SB, C, ST, 8 months	D, community	Un-validated, 30 items	Stigma decreased
Chao, 2010 [75], South Africa, QE/NC	Teachers	120	I, SB, C, CD or 2-day workshop	D, individual	Un-validated, 13 items	Stigma decreased
Gordon-Garofalo, 2004 [83], USA, QE/NC	Family members	28	I, SB, CS, 8 weeks, 2-month F-U	M, interpersonal	Un-validated, 3 items	Stigma decreased
Hosek, 2011 [84], USA, QE/NC	PLHIV	50	I, SB, CS, 12 sessions, 3 months	M, individual	Validated, 40-item scale	Stigma decreased
Lakshmi, 2013 [98], India, QE/C	PLHIV	120	I, SB, CS, 6, 60-minute sessions	M, individual	Validated, 40-item scale	Stigma decreased
Li, 2013 [94], China, RCT	HCWs	1760	I, SB, ST, 1 year, 2 months	D, F, individual, organizational	Un-validated, 30 items	Stigma decreased
Mall, 2013 [78], South Africa, RXS	Community members	1921	I, SB, B, 2 years	D, individual, public policy	Un-validated, NS	Stigma decreased
Nuwaha, 2012 [97], Uganda, RXS	Community members	1402	I, CS, B, 2-year period	D, M, individual, interpersonal, public policy	Validated, 3-item scale	Stigma decreased
Pisal, 2007 [59], India, QE/NC	HCWs	480	I, SB, C, 4 days	D, individual	NS, NS	Stigma decreased, with the exception of comfort cleaning up stool and urine of PLHIV
Uys, 2009 [95], Lesotho, Malawi, South Africa, Swaziland, Tanzania, QE/NC	Setting nurses, team Nurses, PLHIV	134, 43, 41	I, SB, C, 5 days	D, M, individual, organizational	Validated (HASI-P and HASI-N), 52-item scale	Perceived stigma decreased for PLHIV. No change in stigma for nurses
Intervention strategies used						
Gurnani, 2011 [58], India, Programme monitoring data	FSWs, government officials, Police, Journalists	60,000, 175, 13,500 950	SB, CS, C, ST, 4 years	D, M, individual, organizational	Un-validated, 2 items	Stigma decreased
Khuat Thi Hai, 2008 [93], Vietnam, QE/NC	HCWs	1592	I, SB, C, ST, 1-day workshop, 1.5-day training (Arm A), 2-day training (Arm B)	D, F, M, individual, organizational	NS, NS	Stigma decreased
Nyblade, 2008 [96], Vietnam, QE/NC	Community members	2,885	I, SB, C, ST, 1 year, 8 months	D, F, M, Community	Validated and un-validated, 21 items	Stigma decreased
Rao, 2012 [34], USA, QE/NC	PLHIV	24	I, SB, CS, C, 2 days	M, Individual	Validated, 14-item scale	Stigma decreased

a Study design abbreviations: RXS=repeated cross-sectional surveys; QE/NC=quasi-experimental with no control group; QE/C=quasi-experimental with a control group; RCT=randomized controlled trial

b Study population abbreviations: MSM=men who have sex with men; FSW=female sex workers; PLHIV=people living with HIV; HCWs=healthcare workers

c Intervention strategy abbreviations: I=information-based; SB=skills building; CS=counselling/support; C=contact; ST=structural; B=biomedical

d Stigma domain abbreviations: D=drivers; F=facilitators; M=manifestation

e Individual; interpersonal; organizational; community; and public policy

f NS=not specified

* *This study included qualitative data only

### Quality assessment

Two reviewers (JKL and CEH) assessed the quality of quantitative data from studies with randomized controlled trial (RCT), quasi-experimental or mixed-methods study designs (Table 2) using a modified Downs and Black checklist [55]. The checklist consisted of 26 items representing five sub-scales: reporting, external validity, bias, confounding and power [55]. Few of the 48 studies reported power calculations to determine if they had sufficient sample sizes to assess the effectiveness of their interventions. Therefore, we removed the power question (#27) from the standard checklist. The maximum score for the modified checklist was 26. Although the Downs and Black checklist does not have a pre-specified cutoff for acceptable studies, the mid-point score of 13 was used as a guideline to distinguish between low- and high-quality studies [56].

**Table 2:** Quality assessment of the 48 studies

First author, publication date	Study design^a^	Summary score for quality critique
Quantitative (modified Downs and Black, 1998)		
Al-Mazrou, 2005 [92]	QE/NC	62% (16/26)
Apinundecha, 2007 [101]	QE/C	62% (16/26)
Bekele, 2008 [73]	QE/NC	65% (17/26)
Bell, 2008 [72]	RCT	73% (19/26)
Boulay, 2008 [85]	RXS	73% (19/26)
Brown, 2009 [74]	QE/C	58% (15/26)
Chao, 2010 [75]	QE/NC	50% (13/26)
Denison, 2012 [79]	QE/C	50% (13/26)
Deutsch, 2007 [82]	QE/C	65% (17/26)
Esu-Williams, 2004 [77]	QE/C	46% (12/26)
Ezedinachi, 2002 [87]	RCT	58% (15/26)
Fakolade, 2010 [86]	RXS	62% (16/26)
Gordon-Garofalo, 2004 [83]	QE/C	54% (14/26)
Hosek, 2011 [84]	QE/NC	54% (14/26)
Jurgensen, 2013 [80]	RCT	73% (19/26)
Kaponda, 2009 [71]	QE/NC	46% (12/26)
Lakshmi, 2013 [98]	QE/C	50% (13/26)
Lau, 2005 [64]	QE/NC	58% (15/26)
Li, 2010 [60]	RCT	65% (17/26)
Li, 2011 [65]	QE/C	65% (17/26)
Li, 2013 [94]	RCT	73% (19/26)
Mall, 2013 [78]	RXS	58% (15/26)
Maughan-Brown, 2010 [102]	RXS	46% (12/26)
Nambiar, 2011 [69]	QE/C	54% (14/26)
Norr, 2007 [76]	QE/NC	50% (13/26)
Norr, 2012 [91]	QE/C	65% (17/26)
Nuwaha, 2012 [97]	RXS	69% (18/26)
Rao, 2012 [34]	QE/NC	58% (15/26)
Richter, 2012 [103]	QE/NC	46% (12/26)
Rimal, 2008 [70]	RXS	62% (16/26)
Saad, 2012 [88]	RCT	73% (19/26)
Wang, 2009 [61]	QE/NC	42% (11/26)
Williams, 2006 [62]	QE/NC	46% (12/26)
Wu, 2008 [63]	QE/C	62% (16/26)
Yiu, 2010 [68]	QE/NC	77% (20/26)
Young, 2010 [90]	RCT	65% (17/26)
Qualitative (Spencer et al. 2003)		
Biradavolu, 2012 [104]	Qualitative pre- post-	44% (8/18)
Mixed methods (Modified Downs and Black, 1998)		
Adam, 2011 [81]	QE/NC	50% (13/26)
Khuat Thi Hai, 2008 [93]	QE/C	58% (15/26)
Neema, 2012 [99]	RXS	42% (11/26)
Nyblade, 2008 [96]	QE/NC	54% (14/26)
Paxton, 2002 [66]	QE/C	62% (16/26)
Pisal, 2007 [59]	QE/NC	42% (11/26)
Smith Fawzi, 2012 [89]	QE/NC	54% (14/26)
Sorcar, 2009 [67]	QE/C	69% (18/26)
Tshabalala, 2011 [100]	QE/C	54% (14/26)
Uys, 2009 [95]	QE/NC	58% (15/26)
Other		
Gurnani, 2011 [58]	Monitoring data	n/a

a Study design abbreviations: RXS=repeated cross-sectional surveys; QE/NC=quasi-experimental with no control group; QE/C=quasi-experimental with a control group; RCT=randomized controlled trial.

N/a=this study could not be scored using either method as it lacked a research study design and used quantitative program monitoring data only to assess the intervention.

A guide for critically appraising qualitative research was used to appraise the qualitative study [57]. Quality was assessed with 18 items representing nine sub-scales: findings, design, sample, data collection, analysis, reporting, reflexivity and neutrality, ethics and auditability [57]. A score greater than 9, the mid-point for the Spencer guide, was considered high quality. We were unable to assess the quality of one study using either checklist, as the article presented programme monitoring data to assess the structural approach employed [58].

### Data synthesis

Due to the lack of standardized reporting of primary and secondary outcomes, a meta-analysis was not conducted. Instead, we categorized studies by their intervention strategies, and the stigma domains and socio-ecological levels targeted.

Four intervention categories originally described by Brown et al. (2003) were used, including:

1. information-based approaches,
2. skills building,
3. counselling/support, and
4. contact with affected groups.

We included two additional categories: structural approaches and biomedical, to capture new stigma-reduction strategies. Stigma domains assessed were: drivers, facilitators, intersecting stigmas and manifestations [19]. Socio-ecological levels assessed were: individual (knowledge, attitudes, skills); interpersonal (family, friends, social networks); organizational (organizations, social institutions, work place); community (cultural values, norms, attitudes); and public policy (national and local laws and policies) [42].

## Results

**Figure 1:**
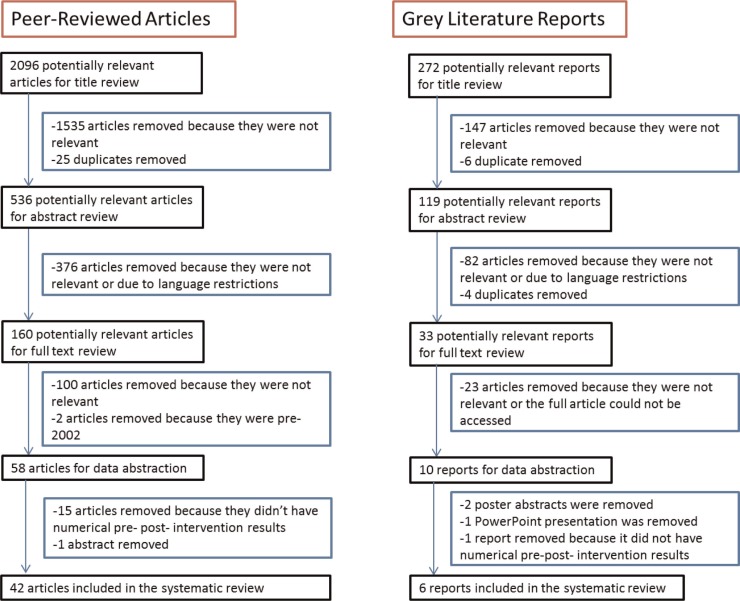
Flowchart of search strategy.

The search criteria identified 4032 potentially relevant articles and reports. After removing 927 duplicates and 737 articles published before 2002, 2096 peer-reviewed articles and 272 grey literature reports were included in the title review phase (Figure 1). A total of 48 (40 peer-reviewed articles, 6 grey literature reports and 2 dissertations) met the inclusion criteria and were included for further analysis.

### Study and intervention characteristics

The studies spanned a large geographical area. Eighteen studies were conducted in the Asia and Pacific region [59–69] and 17 were conducted in the East and Southern Africa [70–80]. Five studies were conducted in North America, Western and Central Europe [34, 81–84] and four were conducted in West and Central Africa [85–88]. Two studies were conducted in Latin America, one study in the Caribbean [89–91] and one study in the Middle East and North Africa [92]. No studies from Eastern Europe and Central Asia were identified. The most represented countries were South Africa (7 studies), China (6 studies), India (6 studies), Malawi (4 studies), and Nigeria (4 studies) (Table 1).

The interventions targeted a wide variety of populations. The most common target populations were students [64–68, 73, 74, 79, 82, 88, 92], healthcare workers [59, 61–63, 71, 87, 91, 93–95], community members [70, 78, 80, 85, 86, 90, 96, 97] and PLHIV [34, 69, 84, 95, 98–101]. Other target populations included youth [72, 77, 102], caregivers [72, 89, 101], teachers [75, 76], market workers [60], family members [83], employees [103] and journalists, police, and community leaders [58, 101]. Three interventions targeted key populations, including female sex workers (FSW) [58, 104] and men who have sex with men (MSM) [81] (Table 1).

**Figure 2:**
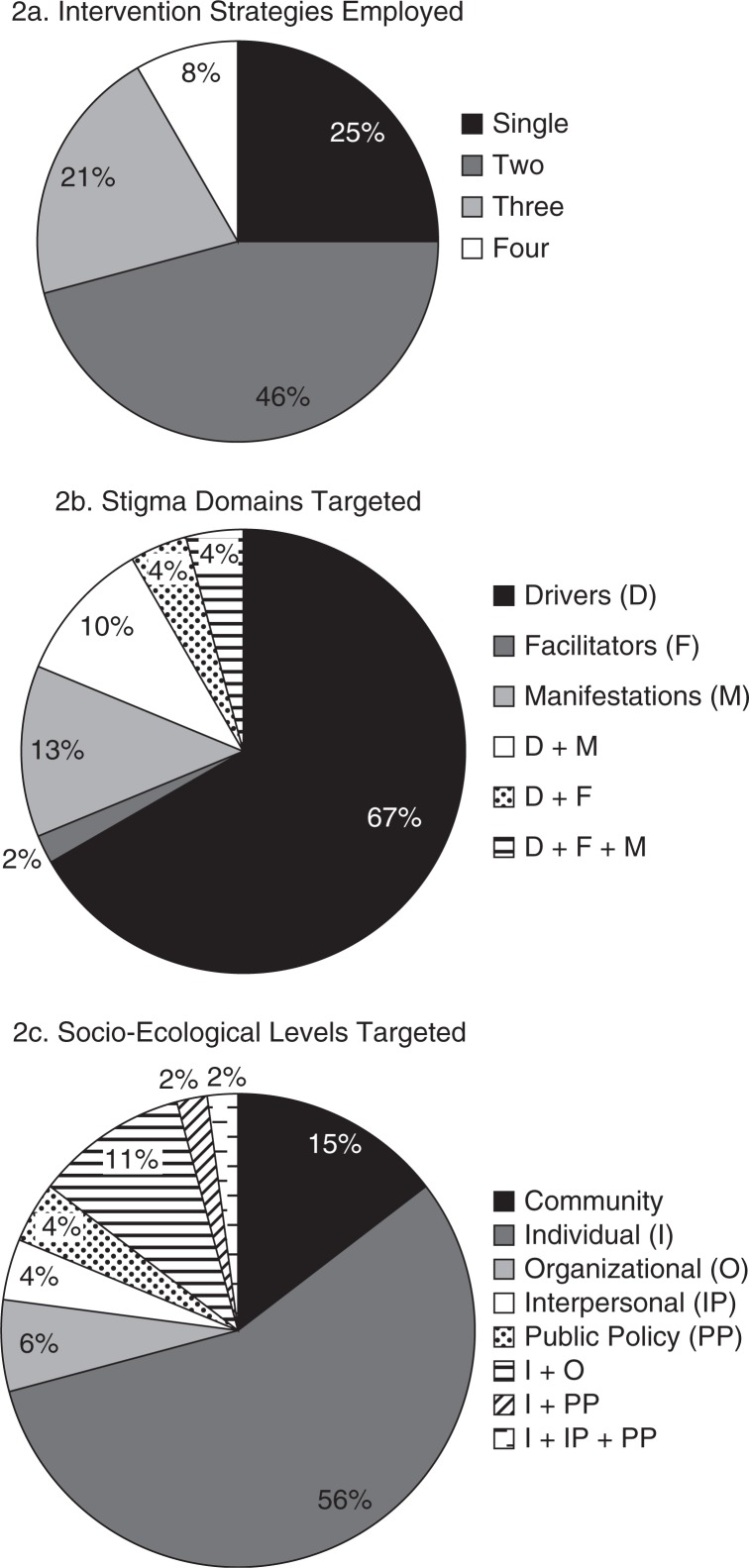
Domains and levels targeted and approaches employed in the 48 studies.

Interventions typically included two or more approaches to reducing HIV-related stigma and discrimination. Forty-six percent used two approaches, 21% used three approaches and 8% used four approaches. However, 12 interventions (27%) employed a single approach (Figure 2a). Information-based approaches were the most common (38 studies), followed by skills-building (32 studies) and contact strategies (14 studies). Only seven studies included counselling/support, six employed structural approaches and four included a biomedical component. All of the studies with a structural component combined it with one or more other intervention strategies [58, 93, 94, 96, 101, 104]. For example, Li et al. combined information and skills building for healthcare workers with provision of universal precaution supplies at intervention hospitals in China [94] and Biradavolu et al. combined skills building and collectivization (into community-based organizations) of FSWs in India [104]. Three of the four studies with a biomedical component also combined it with one or more strategies [78, 80, 97]. For example, Jurgensen et al. and Nuwaha et al. combined community-wide availability of home-based HIV counselling and testing with counselling and support for PLHIV in Zambia [80] and counselling and support and information-based strategies in Uganda [97], respectively. One study assessed a biomedical approach, wider availability of antiretroviral therapy (ART) in South Africa, as a stand-alone stigma-reduction intervention [102] (Table 1).

Most studies (81%) targeted a single stigma domain. Thirty-two studies targeted drivers, one targeted facilitators [99] and six targeted manifestations of the stigmatization process [34, 69, 83, 84, 89, 98]. Only nine studies (19%) targeted multiple stigma domains: five targeted drivers and manifestations [58, 95, 97, 100, 104], two targeted drivers and facilitators [94, 103], and two targeted drivers, facilitators and manifestations [93, 96] (Figure 2b and Table 1). None of the interventions targeted intersecting stigmas.

Forty-one studies (85%) intervened at a single socio-ecological level. Individual-level interventions were the most common (27 studies), followed by community (7 studies), organizational (3 studies), interpersonal (2 studies) and public policy-level (2 studies) interventions. Seven studies targeted multiple levels. The most commonly combined levels were individual and organizational [58, 93–95, 103]. For example, the studies in healthcare settings tended to combine individual-level information provision and/or skills building with organizational-level activities, such as revising hospital policies and/or providing supplies for universal precautions [93–95]. One study by Mall et al. intervened at the individual and public policy levels [78], combining individual-level information and skills building with provision of ART mandated at the public policy level. Finally, the study by Nuwaha et al. targeted the individual, interpersonal and public policy levels [97] (Figure 2c and Table 1).

### Study design and measures

Only 7 of the 48 studies employed a randomized controlled study design [60, 72, 80, 87, 88, 90, 94]. The majority (65%) used quasi-experimental designs either with (13 studies) or without (18 studies) a control group. Another eight used repeated cross-sectional surveys [70, 78, 81, 85, 86, 97, 99, 102], one used programme monitoring data [58] and one used qualitative in-depth interviews collected pre- and post-intervention [104] (Table 1).

The measures used to assess stigma varied considerably across the 47 quantitative studies. Sixteen studies used validated measures, 22 studies (47%) used unvalidated measures or scales, and nine did not specify whether the measures used had been validated previously. Among the 36 studies that described the stigma measures used, only 12 measured the stigma domains that intervention activities were intended to shift. For example, several studies that targeted the drivers of stigma (e.g., fear, prejudice, stereotypes) measured only manifestations of stigma (e.g., agreement with discriminatory statements) [59, 61, 65, 70, 73, 78–80, 86, 91, 99]. The range of items used also differed substantially across studies, with one study using a single measure to assess stigma [65] and one using 61 items [73]. Only two of the seven RCTs reviewed used validated measures [72, 80] and the number of items ranged from 4 to 30 (Table 1).

### Study duration and outcomes

Intervention duration varied widely independent of intervention strategies employed. The shortest intervention tested was a single, 50-minute lecture for nursing students in Hong Kong that employed information-based contact strategies [68]. In contrast, an intervention in Nigeria used the same strategies, but these were implemented over four years [86].

The majority of studies reviewed (79%) reported statistically significant reductions in all stigma measures. Additionally, five studies observed reductions for some stigma measures but not others [59, 69, 70, 95, 100], one study reported reductions for men but not women [90], one reported reductions in both the treatment and control arms [80], and one reported no change in stigma [88]. Only one study in South Africa, which compared discriminatory attitudes reported by young adults in cross-sectional surveys administered before and after ART became widely available in the country, found a significant increase in stigma [102] (Table 1). The biomedical strategy was not combined with any other strategies (e.g., contact, skills building) that have previously demonstrated some effect at reducing stigma [43].

### Quality assessment

Forty-six studies employed quantitative methods and were assessed with the Downs and Black checklist. The average quality score was 15.4 with a median of 15.5. The scores ranged from 11 to 20. The qualitative study was assessed as “low quality” based on the Spencer et al. checklist [104]. Overall, we found the majority of studies to be of high quality, with only nine scoring in the low-quality range. Thirty-seven of the 45 studies (82%) that demonstrated significant reductions in some or all of the stigma measures assessed were considered “high-quality” studies. The study that observed an increase in stigma following the intervention was assessed as a “low-quality” study [102] (Table 2).

## Discussion

This systematic review revealed considerable progress in the stigma-reduction field over the last decade. Yet critical challenges and gaps remain which are impeding the identification of effective stigma and discrimination-reduction strategies that can be implemented by national governments on a larger scale.

### Progress in the field

The number, geography and complexity of interventions studied have expanded considerably. A very high percentage of studies that showed reductions in stigma were of high quality, which is a marked improvement from previous reviews [43, 44, 105]. There has been a substantial shift in the geography of stigma-reduction research. The interventions summarized in our review were conducted predominantly in low- and middle-income countries and targeted a much wider variety of populations. Only 5 of the 48 studies were conducted in the North America, Western and Central Europe region [34, 81–84]. The populations targeted with stigma and discrimination-reduction interventions have also expanded in the past decade. While students and healthcare workers continue to be heavily studied populations, studies among community members [70, 78, 80, 85, 86, 90, 96, 97] and PLHIV [34, 69, 84, 89, 98–100] are becoming more common.

Our review demonstrated that the socio-ecological levels targeted by stigma-reduction interventions have expanded over the past decade to include all five levels of influence. While individual-level interventions remained the most common, several community-level efforts have been tested [60, 70, 85, 86, 90, 96, 101] and a few interventions at the organizational-level have been studied [79, 99, 104]. In addition, interventions targeting multiple socio-ecological levels are beginning to emerge [58, 78, 93–95, 97, 103]. The stigma domains targeted have also expanded to include the facilitators [99] and manifestations of stigma [34, 69, 83, 84, 89, 98] as well as the drivers, sometimes in combination [58, 93–97, 100, 103, 107].

These findings are encouraging, given recent conceptualizations of the stigmatization process that highlight the importance of combining multiple intervention strategies to address multiple stigma domains across multiple socio-ecological levels [36, 52].

### Challenges and gaps

#### Intervention

Despite these improvements, most of the 48 studies targeted a single domain of stigma (drivers) and a single socio-ecological level (individual-level). While these studies provide important insights about potential strategies for improving the attitudes of a variety of individuals and groups (e.g., youth, healthcare workers, employees, students), they do not adequately address stigma manifestations, such as shame and discrimination, or community-level attitudes and social norms that shape individuals’ attitudes and behaviours. This finding calls into question the longer term utility of the interventions described for interrupting the stigmatization process.

Individual-level drivers of stigma, such as knowledge, fear and attitudes, are only part of the stigmatization process. Also critical to address are individual-level manifestations of stigma, such as the anticipation of experiencing stigma if positive or the perception that stigma towards PLHIV is high in a given community, which prevent people from testing for HIV or disclosing their HIV-positive status to a sexual partner or family member [1, 106]. Interventions that fail to address these concerns are unlikely to lead to increased and sustained health seeking behaviour or inspire the adoption of preventive behaviours, two of the key goals of stigma-reduction interventions.

Rigorous evaluations of multi-faceted interventions, designed to target the individual-level manifestations and drivers of stigma, are needed to inform the most efficacious and effective approaches for achieving longer term health outcomes. In addition, more research is needed to explore the individual and combinations of strategies that are most effective at improving community attitudes and creating an enabling environment for PLHIV and key populations to engage with healthcare and social support systems.

There are limited data assessing the influence of stigma-reduction interventions on key behavioural and biomedical outcomes, such as uptake of and retention on ART, drug regimens and feeding practices to prevent vertical transmission, and vertical transmission itself. While stigma is commonly cited as a barrier to prevention efforts [12, 53, 107], and many prevention trials have collected measures of stigma and discrimination [108], no fully powered RCT or quasi-experimental trial of HIV-prevention strategies or technologies have included stigma reduction as a key component of the intervention tested. Given emerging challenges with adherence to drug-based prevention among groups most at risk of HIV infection [109], such data are needed to inform appropriate national responses to the HIV epidemic.

Another gap is the absence of tested interventions aimed at supporting PLHIV to fulfil their human rights to care and dignity. Many countries have expanded existing laws or adopted new ones that protect PLHIV against discrimination [110]. However, for PLHIV to access their rights, they must be aware of the law and be able to access systems of redress against violations of those rights. Legal education and legal aid services are often needed to support PLHIV to access justice, and such services are recommended by UNAIDS as critical [49, 51]. Evaluation data are needed to inform the wider use of such approaches to support the positive advances that have been made in the public policy arena in many countries over the last decade.

Interventions specifically designed to reduce the intersecting stigmas that key populations often face were also absent from the literature. Such strategies will be important for maximizing the participation of key populations in biomedical prevention efforts such as universal HIV testing and treatment and topical and oral chemoprophylaxis with ART [16]. More information is needed on successful strategies to reduce intersecting stigmas in contexts where epidemics are concentrated in key populations, as well as where HIV epidemics among key populations are happening in the context of widespread generalized epidemics [111].

#### Methodology

Evaluating structural stigma-reduction interventions, particularly those targeted at the community level, poses a methodological challenge. Such interventions often involve multiple components occurring simultaneously at multiple levels, and thus are not necessarily conducive to the classic RCT design [112]. In addition, the social norm changes desired typically take longer to achieve than individual-level attitude changes [113]. Three of the studies evaluating interventions with a structural component in this review used quasi-experimental designs [93, 96, 114], one used pre- and post-in-depth interviews [104] and one reviewed programme monitoring data collected during the intervention period [58].

While these studies suggested some positive effects of structural approaches, causality is difficult to establish with these study designs in addition to the difficulties in attributing the relative effectiveness of structural approaches, as compared to the other components of the intervention. Additional research and the development of alternative or new evaluation methodologies such as propensity scores, causal inference and structural equation modelling are needed, particularly given the recent emphasis on addressing the structural causes of stigma and discrimination [115].

#### Measurement

Measurement issues continue to pose an important challenge to the field. The lack of standardized outcome measures for stigma and discrimination greatly limits our collective ability to determine which strategies work the best for addressing the various stigma domains or targeting different socio-ecological levels. While some validated scales have been developed for specific types of stigma, populations and contexts [116–120], few scales demonstrating validity in multiple contexts or across multiple populations are available [121, 122].

A priority moving forward must be the development of validated measures assessing each domain of the stigmatization process that can be shifted with programmatic efforts and/or structural interventions. An instrument similar to the MOS-HIV, which measures multiple domains of health-related quality of life, is validated for use in multiple countries and has standardized instructions for cultural adaptation [123], would greatly enhance the field of HIV stigma research. While some aspects of stigma may be culturally specific, key underlying constructs are common across contexts [24, 29], facilitating the development of standardized measurement tools. Such instruments are needed for assessing stigma and discrimination in the general population, among family and peers, among PLHIV and key populations and among healthcare workers [23, 24, 29]. The standardized survey for use in health facilities presented by Nyblade et al. in this supplement is an encouraging development. Similar efforts are now needed for other populations.

The discordance between the targeted domains of stigma and the measured domains of stigma is of concern. Across the studies reviewed, it was common for intervention activities to target drivers of stigma among individuals (e.g., fear of HIV infection through casual transmission) but only measure stigma manifestations (e.g., agreement with discriminatory statements like “teachers living with HIV should no longer be allowed to teach”) to assess intervention effectiveness [59, 61, 65, 70, 73, 78–80, 86, 91, 99]. This discordance adds another layer of uncertainty to the study findings. Let us take as an example an intervention that is successful at increasing awareness of stigma and its harmful consequences, but not at reducing fear of HIV infection through casual contact, which tends to drive avoidance behaviours. If the researcher only measures willingness to sit next to someone living with HIV and finds no significant change following intervention, she may mistakenly conclude that the intervention was not successful. The field would benefit considerably from evaluations that clearly link the stigma domains being targeted with the stigma domains measured [19]. The development of a uniform conceptualization of the stigmatization process, based on empirical evidence, could inform the development of both interventions and measurement tools.

### Limitations

There are several limitations with the approach used here. We were not able to explore the potential influence of stigma and discrimination-reduction efforts generated from and implemented by communities of PLHIV and key populations, which have been a hallmark of the HIV response in many countries, due to the lack of evaluation data on these approaches in the peer-reviewed and grey literature. Inclusion criteria limiting studies to those with pre- and post-intervention data excluded studies that only used post-intervention data to compare intervention and control groups. However, it was far more difficult to assess these studies’ quality thus limiting the utility of their inclusion for this review. Assessing study quality using the Downs and Black checklist was challenging due to the nature of most stigma-reduction interventions, precluding typical trial components such as blinding. Despite these challenges, the majority of studies reviewed were assessed as being of high quality.

A meta-analysis was not completed due to the significant heterogeneity of interventions and outcomes limiting the assessment of pooled effectiveness of interventions at reducing HIV-related stigma and discrimination. Generalizability of the findings of these interventions is limited as they have been tested only in specific sub-populations, such as students or healthcare workers. Assessment of causality of these interventions was also limited since more than half of the studies did not include a control group. Finally, some studies used un-validated scales or did not list the measurements used, which may lead to uncertainties in the reliability and validity of their measurements. Even with specific inclusion criteria and these limitations, this review draws strength from harnessing nearly 50 studies focused on the mitigation of HIV-related stigma and discrimination representing several types of interventions and populations.

## Conclusions

The field has come far in the last decade, though much remains to be done to enable the integration of proven stigma and discrimination-reduction strategies into national AIDS responses. Complex problems require complex solutions. The field of HIV-prevention research needs to embrace the importance of stigma in the HIV response, rather than shy away from it. The field must become bolder in the design and evaluation of interventions that target multiple stigma domains at multiple levels. Similarly, funding agencies should support the rigorous evaluation of multi-faceted stigma-reduction interventions, including interventions that assess the influence of stigma on behavioural and biomedical outcomes. Our collective ability to translate efficacious biomedical prevention approaches, such as ART as prevention [124–127], into effective ones at the population-level rests on whether we can remove the social and structural barriers to uptake and adherence. As such, addressing HIV-related stigma and discrimination should be at the core of the HIV response, not at the fringes. This priority should be represented in funding, policy, research and programming.
